# Unexpected Difficult Airway Management in a Grossly Malformed Newborn Baby With Tessier Cleft After an Emergency Cesarean Section

**DOI:** 10.7759/cureus.26832

**Published:** 2022-07-13

**Authors:** Shipra Tandon, Mridul Dhar, Amrita Bannerjee

**Affiliations:** 1 Department of Anesthesiology, All India Institute of Medical Sciences, Rishikesh, IND

**Keywords:** difficult airway management, neonatal airway management, neonatal airway, tessier cleft, congenital anomaly, congenital malformation, difficult airway

## Abstract

Emergent obstetric deliveries may present the anesthesiologist with a unique challenge of managing the airway of previously undiagnosed syndromic or malformed newborns in the obstetric theatre. The present report describes an emergency cesarean section in a 32-year-old lady who delivered a newborn with grossly anomalous features, the challenges in airway management in the newborn, and a discussion on preparation and sensitization about encountering such scenarios for the anesthesiologist and the associated medical teams.

## Introduction

Enhanced perinatal obstetric care and increased scope and coverage of these services in peripheral and remote regions have led to earlier recognition of congenital anomalies and timely termination if indicated. However, often un-booked cases end up in hospitals at the time of delivery or in labor in an emergent setting [1]. This may pose a unique challenge to the treating physician in terms of airway management in a newborn with malformed facial features. A case of airway management is described in a newborn baby with bilateral Tessier cleft and hydrocephalus following an emergency cesarean section.

## Case presentation

An emergency cesarean section was scheduled for a 32-year-old, gravida 3, para 2 + 1 patient with a history of consanguineous marriage and a previous cesarean section because of scar tenderness. This was an un-booked case with no investigations including antenatal ultrasound. The pre-anesthetic evaluation revealed no major issues in the mother (American Society of Anesthesiologists physical status class II-E). The patient was taken for surgery and was given 2.2 mL (11 mg) of 0.5% injection of hyperbaric bupivacaine via spinal anesthesia in the left lateral decubitus position. The baby was delivered within five minutes of the lower abdomen transverse incision and was handed over to the neonatologist receiving the baby.

**Figure 1:**
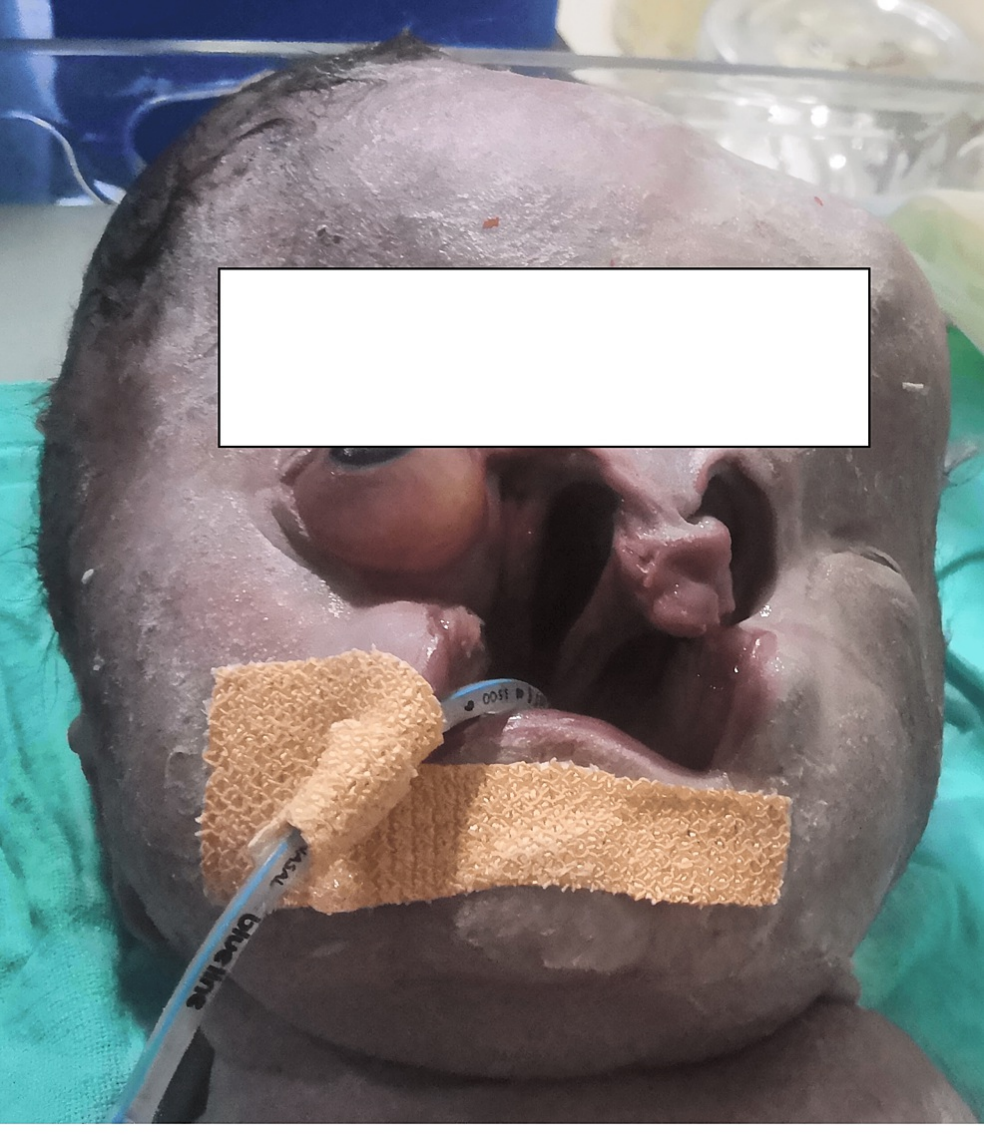
Grossly anomalous craniofacial features of the newborn baby

The delivered newborn appeared to have grossly anomalous features and required urgent airway management by the neonatology team. On visual appearance, the neonate had an exposed bulging left eye and severe hydrocephalus (Figure 1). Nasal and oral structures could not be appreciated. Vitals signs were heart rate of 68/minute and oxygen saturation of 85%, with an APGAR (appearance, pulse, grimace, activity, and respiration) score of 3. Because of such distorted anatomy, the assistance of the anesthesiology team was sought. Conventional bag-mask ventilation was not possible due to the facial defect. A larger mask covering the whole orifice of the defect was attempted with minimal success but was able to sustain the oxygen saturation to around 90%. Direct laryngoscopy with a Miller straight blade (size 1) was attempted first with supplemental oxygen given via a catheter resting in the oropharyngeal area, but nothing could be visualized as the blade put pressure on the eyeball directly, impeding proper laryngoscopy and glottic vision. The smallest available supraglottic airway (SGA) device was attempted (i-gel® size 1, Intersurgical Ltd., Wokingham, UK) but it failed to provide any seal to transmit the ventilation. While further expert help was sought with the arrangement of surgical airway access, another attempt of laryngoscopy was done with a Macintosh curved blade (size 1), which was fortunately successful in inserting the endotracheal tube (3 mm internal diameter) into what appeared to be the glottic opening. Tube position was confirmed by chest rise and auscultation. The baby was transferred to the neonatal intensive care unit for further management but did not ultimately survive because of the gross congenital anomalies and possible associated cardiac defects. The diagnosis of the craniofacial defect established later was Tessier cleft 3 on the left and 3, 4, and 6 on the right side (Figure 1).

## Discussion

Newborns with facial clefts can present with varying levels of airway difficulty based on the type and extent of the deformity [2,3]. In cases such as the present case, it is difficult to identify the correct location of the glottis while performing conventional laryngoscopy, as the nearby anatomical relations like the uvula, tongue, palate, and epiglottis may also be distorted. In this case, the exposed eyeball also impeded the correct placement of the laryngoscope blade. During airway management of newborns, the primary technique is often conventional Miller blade laryngoscopy, even if the airway is considered difficult [2,4]. If unsuccessful, alternate techniques like video laryngoscope (if available) and SGA insertion can be attempted with the aim to maintain oxygenation and ventilation [4]. Although in the present case SGA failed to provide adequate seal and ventilation, it is an invaluable device in such cases. Invasive surgical access to the front of the neck and specialist otorhinolaryngology help should be sought as a last resort rescue technique if all other methods fail [2,5].

In routine elective operative cases involving neonates and older children, the anesthesiologist has an opportunity to assess the airway, devise the airway management plan, and arrange the associated equipment. In the present case, the airway management in addition to being difficult was also relatively unexpected, as prior details of the baby’s syndromic condition were not known. In this context, it is perhaps prudent to have neonatal difficult airway equipment and cricothyroidotomy options on standby in obstetric operation theatres even if not used routinely [6]. This is important, especially for centers that often get emergent cases with no prepartum evaluation of the baby’s condition.

Teamwork and good communication with the obstetric and the neonatology team are very essential to have a favorable outcome in such cases, especially in this scenario where the anesthesiologist has to take care of the mother under anesthesia as well as assist the pediatrician/neonatologist in airway management of the newborn baby [4]. Intermittent training and debriefing of teams working in the obstetric theatre should be performed to sensitize them regarding encountering such scenarios and increase awareness of similar possibilities in syndromic newborns with other craniofacial anomalies associated with respiratory distress [2,5]. This should help in better team dynamics and coordination during the time of an acute airway emergency.

## Conclusions

Anesthesiologists involved in obstetric anesthesia might encounter such emergent cases rarely and could be relatively unprepared for difficult airway management. The neonate in the current case in most likelihood was not compatible with life and would have had a poor outcome irrespective of airway management. Anesthesiologists and other team members involved in obstetric cases should strive to be adequately prepared to encounter such cases and manage the airway of syndromic or malformed babies with a coordinated team approach.

## References

[1] Tucker A, Ogutu D, Yoong W, Nauta M, Fakokunde A (2010). The unbooked mother: a cohort study of maternal and foetal outcomes in a North London hospital. Arch Gynecol Obstet.

[2] Infosino A (2002). Pediatric upper airway and congenital anomalies. Anesthesiol Clin North Am.

[3] Mazzola RF, Mazzola IC (2014). Facial clefts and facial dysplasia: revisiting the classification. J Craniofac Surg.

[4] Krishna SG, Bryant JF, Tobias JD (2018). Management of the difficult airway in the pediatric patient. J Pediatr Intensive Care.

[5] Hernández-Cortez E, Martinez-Bernal GF (2016). Airway in the newborn patient. J Anesth Crit Care.

[6] Johansen LC, Mupanemunda RH, Danha RF (2012). Managing the newborn infant with a difficult airway. Infant.

